# Early onset and liver failure indicating poor prognosis of infant liver failure syndrome type 1

**DOI:** 10.1186/s13023-024-03229-3

**Published:** 2024-06-06

**Authors:** Shu-Yuan Li, Jia-Yan Feng, Zhong-Die Li, Teng Liu

**Affiliations:** 1 Department of Hepatology, The Center for Pediatric Liver Diseases, Children’s Hospital of Fudan University, 399 Wanyuan Road, Shanghai, 201102 China; 2 The Department of Pathology, Children’s Hospital of Fudan University, Shanghai, 201102 China

**Keywords:** Acute liver failure, Genotype, Survival analysis, *LARS1*, Leucyl-tRNA synthase 1

## Abstract

**Background:**

Infantile liver failure syndrome type 1 (ILFS1, OMIM #615,438), caused by leucyl-tRNA synthase 1 (*LARS1*, OMIM *151,350) deficiency, is a rare autosomal-recessive disorder. The clinical manifestations, molecular-genetic features, and prognosis of *LARS1* disease remain largely elusive.

**Methods:**

Three new instances of ILFS1 with confirmed variants in *LARS1*, encoding LARS1, were identified. Disease characteristics were summarized together with those of 33 reported cases. Kaplan-Meier analysis was performed to assess prognostic factors in ILFS1 patients.

**Results:**

The 3 new ILFS1 patients harbored 6 novel variants in *LARS1*. Among the 36 known patients, 12 died or underwent liver transplantation. The main clinical features of ILFS1 were intrauterine growth restriction (31/32 patients in whom this finding was specifically described), failure to thrive (30/31), hypoalbuminemia (32/32), microcytic anemia (32/33), acute liver failure (24/34), neurodevelopmental delay (25/30), seizures (22/29), and muscular hypotonia (13/27). No significant correlations were observed between genotype and either presence of liver failure or clinical severity of disease. Kaplan-Meier analysis indicated that age of onset < 3mo (*p* = 0.0015, hazard ratio = 12.29, 95% confidence interval [CI] = 3.74–40.3), like liver failure (*p* = 0.0343, hazard ratio = 6.57, 95% CI = 1.96-22.0), conferred poor prognosis.

**Conclusions:**

Early age of presentation, like liver failure, confers poor prognosis in ILFS1. Genotype-phenotype correlations remain to be established.

**Supplementary Information:**

The online version contains supplementary material available at 10.1186/s13023-024-03229-3.

## Background

Infantile liver failure syndrome type 1 (ILFS1, OMIM #615,438) is an autosomal recessive disorder associated with variants in *LARS1* (OMIM *151,350), encoding leucyl-tRNA synthetase 1 (LARS1), with dysfunction of protein polypeptide synthesis and glucose sensing [1]. LARS1 is responsible for correct coupling of leucine to cognate tRNAs during protein polypeptide synthesis and plays a role in interactions between glucose sensing and leucine metabolism [2]. The phenotype of ILFS1 is heterogeneous, with multisystem involvement that includes severe intrauterine growth retardation (IUGR), microcytic anaemia, fulminant liver failure, and more. However, as reported cases are few, correlation of clinical, histopathologic, and genetic features with prognosis of *LARS1* disease is incomplete [3].

This study describes 3 new patients with ILFS1 and reviews identifiable reports of ILFS1 with confirmed LARS1 deficiency. It summarizes the principal manifestations of *LARS1* disease and evaluates factors that may signal prognosis.

## Methods

Three children were admitted to our centre due to cholestasis that was suspected to be caused by LARS1 deficiency after whole genome sequence as per the guidelines of American College of Medical Genetics/Association for Molecular Pathology guidelines [4]. The diagnosis ruled out other potential causes of cholestasis such as infection, hemolytic jaundice, endocrine disease, bile acid synthesis disorders and other metabolic conditions after conducting appropriate investigations. All 3 children were enrolled in this study with parental consent, under a protocol approved by the Children’s Hospital of Fudan University and in compliance with the ethical guidelines outlined in the 1975 Declaration of Helsinki.

The 3 patients’ records were analysed alongside those in published case reports, all of which described confirmed *LARS1* recessive variants (except one patient with no detailed information of *LARS1* variants). The analysed demographic data included the child’s gender, age of clinical disease onset, and the clinical signs such as anaemia, liver involvement, kidney involvement, muscle involvement as well as nervous involvement. These pieces of information were used to assess patients’ status during follow-up and to evaluate the main factors that might reflect prognosis.

### Pathogenicity of newly discovered missense *LARS1* variants

Predicted pathogenicity of novel missense variants was assessed by the in silico tools Mutation Taster (http://www.mutationtaster.org/), Sorting Intolerant From Tolerant (SIFT, http://sift.jcvi.org), Protein Variation Effect Analyzer (PROVEAN, http://provean.jcvi.org/index.php), Mendelian Clinically Applicable Pathogenicity (http://bejerano.stanford.edu/MCAP/), Functional Analysis Through Hidden Markov Models (http://fathmm.biocompute.org.uk/), and Rare Exome Variant Ensemble Learner (https://sites.google.com/site/revelgenomics/). All were used with default settings.

### Liver histopathology

Liver-biopsy specimens were fixed in formalin, routinely embedded in paraffin, and sectioned at 4 μm. Tissue sections picked up on glass slides were stained, using standard procedures, with hematoxylin and eosin, with periodic acid – Schiff technique, with Masson trichrome technique, for iron, and for reticulin fibres. Light microscopy was undertaken.

### Statistical analysis

Statistical analysis using IBM SPSS 19.0 software (IBM Corporation, Somers, NY, USA) determined differences in parameters between patients with different clinical outcomes. Student’s t-test was performed when data showed a normal distribution. Nonparametric Mann-Whitney U testing was performed when data did not show a normal distribution. Data are expressed as mean ± standard deviation or median (interquartile range, IQR). Receiver operating characteristic curve analysis was used to calculate the area under the curve. Fisher’s exact test is used in “R×C table”. Kaplan-Meier plots were used to assess factors potentially associated with prognosis. A *p* value < 0.05 was considered statistically significant.

## Results

### Clinical histories and LARS1 status, 3 patients

Patient 1 was a Han boy born at 32 + 2 weeks of gestation and with a weight of 1150 g at birth. He is the first child of a Chinese couple. His mother was 28 years old during pregnancy and had no history of spontaneous abortion or any genetic diseases. She has received progesterone treatment for one month due to low levels of human chorionic gonadotropin and progesterone during the first trimester. The boy was admitted to the neonatal care unit due to his premature delivery and shortness of breath. He was also observed to have jaundice on the second day after birth. Irreversible severe hypoalbuminemia (minimum 23 g/L) and anemia (minimum 52 g/L) were observed as well after repeated infusion of red cell suspension and albumin (Table 1). Jaundice continued to be present despite receiving treatments including nutrition support, continuous positive airway pressure, antibiotics, ursodeoxycholic acid, and fat-soluble vitamin supplementation. The boy was admitted to the local centre because he was experiencing persistent jaundice and failure to thrive. When he was sent to us, he was 5 months and 12 days old with the corrected gestational age being 3 months and 18 days. The boy had a circumference of the head 37 cm (< percentile (P) 1), length 49 cm (< *P*1), weight 4.0 kg (< *P*1). He fell behind in the development of the nervous system as the boy could only raise his head for 1 min, but couldn’t turn over. The physical examination indicated a chubby face (Supplement Fig. 1.A&B) with moderate to severe jaundice. Abdominal distension was noticed with hepatosplenomegaly (liver edge 4 cm and spleen tip 2 cm below costal margin). No abnormality was identified on specialist neurological examination.

**Table 1:** Results, laboratory testing

	P1	P2	P3	NRM
Age	5.0 m	0.3 m	2.0 m	
WBC (×10^9^/L)	4.5–27.3	10.1–25.0	6.8–7.1	5.0–12.0
HB (g/L)	52.0-143.0	82.0-199.0	77.0-104.0	99.0-196.0
MCV (fL)	65.0–87.0	86.0-100.0	79.0–89.0	73.0-105.0
MCH (pg)	18.5–30.0	30.0–34.0	26.0–31.0	24.0–37.0
MCHC (g/L)	285.0-346.0	311.0-366.0	316.0-357.0	305.0-361.0
PLT (×10^12^/L)	57.0-502.0	120.0-280.0	111.0-214.0	100.0-300.0
RET (%)	1.0-6.9	0.1–1.2	0.4–0.7	0.5–1.5
Alb (g/L)	18.3–23.0	18.0–31.0	20.0–32.0	35.0–50.0
ALT (IU/L)	3.0-196.9	42.0–84.0	21.0-113.0	8.0–71.0
AST (IU/L)	22.0-441.9	32.0-118.0	41.0-306.0	21.0–80.0
GGT (IU/L)	185.0–42.0	158.0–44.0	33.0–45.0	9.0-150.0
TB (µmol/L)	21.7-187.3	113.0-299.0	195.0-414.0	3.4–17.1
DB (µmol/L)	7.8-122.2	67.0-276.0	133.0-273.0	0.0–6.0
TBA (µmol/L)	92.1-278.4	130.0-200.0	204.0-347.0	0.0–10.0
CK (IU/L)	-	81.0-157.0	74.0	24.0-170.0
INR	2.1–2.4	1.5–2.6	1.8–2.6	0.8–1.2
Ammonia (umol/L)	43.6	-	111.0	18.0–72.0
Glycemia (mmol/L)	3.9	2.4–5.9	4.2–5.3	3.9–6.1
Ferritin (ng/mL)	1328.0	1543.0	>2000.0	26.0-287.0

*Abbreviations*: *P* Patient, *NRM* Normal reference range, *WBC* White blood cells, *HB* Hemoglobin, *PLT* Platelets, *RET* Percentage of reticulocyte, *ALT* Alanine aminotransferase, *AST* Aspartate aminotransferase, *GGT* Gamma-glutamyltransferase, *TB* Total bilirubin, *DB* Direct bilirubin, *TBA* Total bile acid, - not available, *ALB* Albumin, *CK* Creatine kinase, *INR* International normalized ratio

The laboratory examination revealed severe microcytic hypochromic anemia (minimum 52 g/L, mean corpuscular volume of 65-87fL, mean corpuscular hemoglobin of 18.5-30pg, Mean corpuscular hemoglobin concentration 285–346 g/L, reticulocyte 1-6.9%) after bone marrow cytology. No abnormalities were observed in serum iron, unsaturated iron binding capacity, total iron binding capacity, transferrin saturation, thalassemia gene, G6PD enzyme activity, folic acid, and Vitamin B12 levels. and Coombs test, except for ferritin at 1328ng/ml (normal reference range, NRM: 26-287ng/mL). Hepatobiliary-system biomarker values included total bilirubin (TB) 21.7-187.3umol/L, direct bilirubin (DB) 7.8-122.2umol/L, alanine aminotransferase (ALT) 3-196.9U/L, aspartate aminotransferase (AST) 22-441.9U/L, total bile acids (TBA) 92.1-278.4umol/L, progressive declined gamma-glutamyl transferase (GGT) level (185-42U/L) with low albumin (a minimum of 18.3 g/L), coagulation abnormality (international normalized ratio, INR 2.1–2.4, after vitamin K1 injection), with normal ammonia. The level of alpha-fetoprotein was 19665ng/mL (NRM: <28ng/mL). Blood analysis by tandem mass spectrometry showed elevated levels of tyrosine, methionine, and arginine during liver failure. No abnormalities were observed on TORCH or hepatitis-virus serologic studies, on blood and urine culture, or in values for fasting cortisol and adrenocorticotropic-hormone levels, biomarkers of thyroid function, immunoglobulins, autoantibodies, lymphocyte subsets, urinary organic acids, fasting blood glucose, blood ketones, and lipids.

Computerised tomograms of the thorax (Supplement Fig. 1. C**-**F) showed exudation in both lungs with decreased thoracic bone density. Findings on brain magnetic-resonance imaging suggested myelination less than in full-term children of the same postnatal age. Whole exome sequencing indicated biallelic variation in *LARS1* [c.1284G > A (p.Pro428Pro); c.3379 C > T (p.Arg1127Ter)]. The patient’s clinical signs improved, with resolution of liver failure, after infusion of red blood cell suspension, albumin, immunoglobulin, and cefepime as well as with nutritional support, ursodeoxycholic acid (UDCA), and fat-soluble vitamins. However, after discharge from hospital at 6.5 months, the boy’s liver function deteriorated sharply with severe hypoproteinemia and severe anemia after development of fever and cough. He died in multiple organ failure aged 8.4 month.

Patient 2 was a boy, the second child of a non-consanguineous couple who had no remarkable medical history. He was born full-term, at 39 weeks and 2 days with a normal birth weight (3700 g). He developed diarrhea shortly after birth and was transferred to our center because of aggravated cholestasis. At 34 days of age, laparoscopic biliary exploration was performed to exclude biliary atresia due to the presence of alcoholic stools. The liver function progressively worsened, with coagulopathy (INR 2.62), anemia (minimum 82 g/L), hypoalbuminemia (minimum 18 g/L), and hypoglycemia (2.4mmol/L). Whole exome sequencing indicated biallelic variation in the *LARS1* gene [c.1321 C > T (p.Arg441Ter); c.149 C > G (p.Ala50Gly)]. Nutrition supplement support and UDCA treatment. The boy died at home (at parental request) in liver failure, with unresolved diarrhea, aged 2 month.

Patient 3 is a female. She was born prematurely at 35 weeks and 2 days of gestation to a non-consanguineous couple with a low birth weight of 1400 g (< *P*3). Her mother experienced gestational hypertension during pregnancy, and following birth, the patient experienced asphyxia and rescue. The patient was noted to have jaundice on the second day after birth. Increased levels of direct bilirubin were observed on the ninth day after birth. An increase in transaminase levels was noted until one month of age. The child was diagnosed with anemia (minimum 77 g/L), hypoalbuminemia (minimum 20 g/L), coagulopathy (INR = 2.62) and hyperammonemia (111umol/L). Physical examination found hepatomegaly. Elevated citrulline was found by blood analysis using tandem mass spectrometry. Whole exome sequencing indicated biallelic variation in the *LARS1* gene [c.497T > C (p.Leu166Pro); c.2806T > C (p.Cys936Arg)]. The patient’s condition greatly improved and she had been discharged from the hospital after receiving anti-infection treatment, infusion of red blood cell suspension, albumin, immunoglobulin, and nutritional support. Additionally, UDCA and fat-soluble vitamins were also administered during the hospital stay. However, the girl died at age of 6 month in acute liver failure associated with fever.

### Literature review

**Fig. 1:**
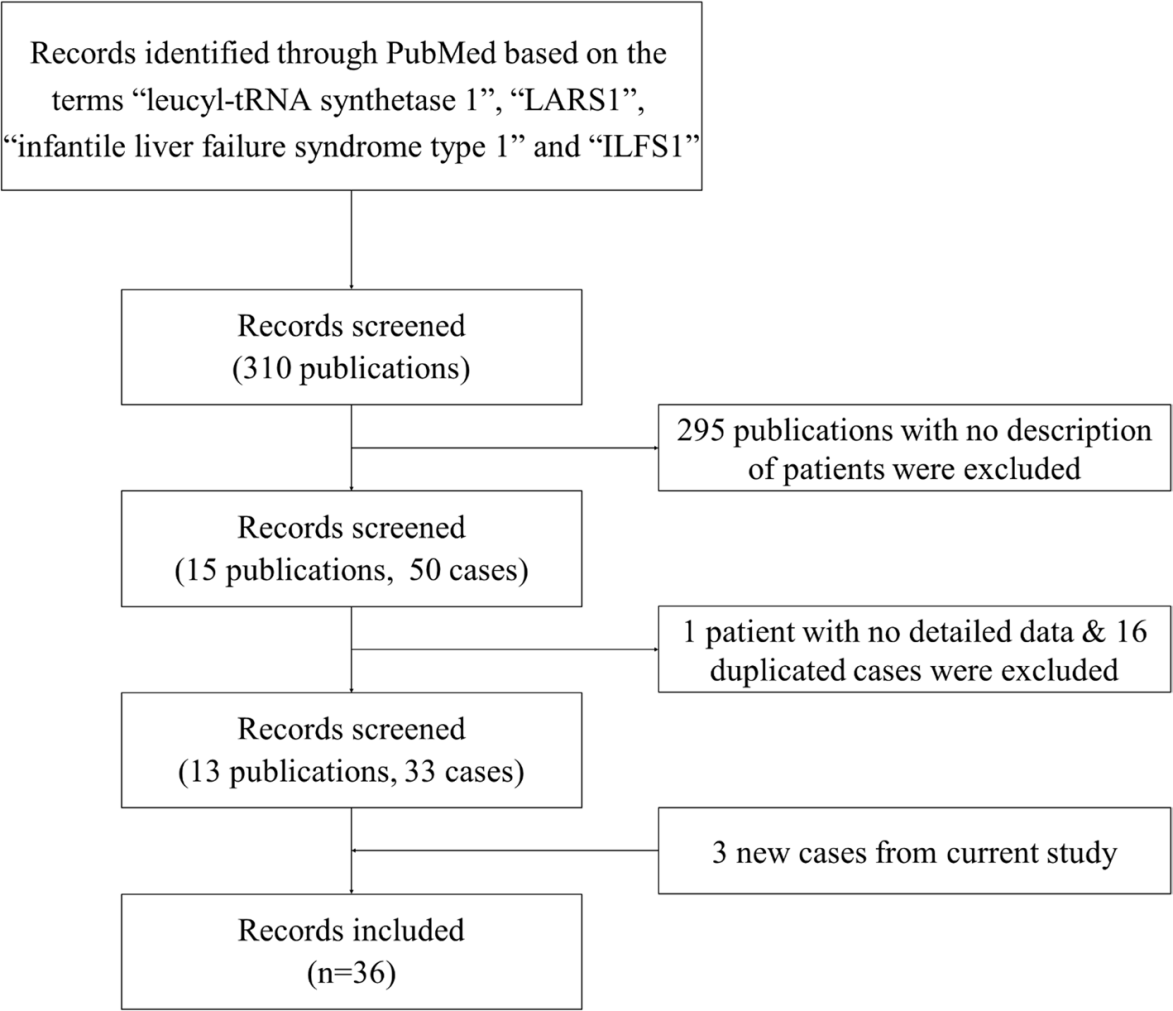
Diagram of screening and selection of included cases

The algorithm employed is schematized in Fig. 1. PubMed (https://pubmed.ncbi.nlm.nih.gov/) was searched for the terms “leucyl-tRNA synthetase 1”, “*LARS1*”, “infantile liver failure syndrome type 1”, and “ILFS1”. Descriptions of patients with confirmed *LARS1* variants were collected. In all, 33 [1, 4, 6–16] cases from 14 publications were identified. Clinical manifestations, laboratory values, histopathologic findings in liver, and LARS1 status were reviewed.

### Clinical features of reported ILFS1 patients

Among the 36 patients enrolled, 22 were male and 13 were female; gender in one was not specified (Table 2). IUGR was noted in 31 patients (31/32). 14 of 23 patients were reported to have had premature delivery. The ages of disease onset ranged from 0 day after birth to 23 years, with a median age of 2 months. 31 of 32 patients had been diagnosed with IUGR.

**Table 2:** Demographics and extrahepatic clinical manifestations of *LARS1* disease in 36 ILFS1 patients

Basic information			Birth		Growth	Blood	Nervous system			Renal system			Muscle	Others		Status at last follow-up	Ref
ID	G	age of onset	premature delivery	IUGR	FTT	anemia	neurodevelopmental delay	encephalopathic crisis	seizures	renal calculus	renal failure	renal tubular injury	hypotonia / skeletal muscle abnormality	hypoalbuminemia	hypoglycemia		
1	M	2 m	/	+	+	+	+	-	+	-	-	-	-	+	-	Alive at 12y	[16]
2	F	2 m	/	+	+	+	+	-	+	-	-	-	-	+	-	Alive at 3y	
3	M	< 1y	/	+	+	+	+	-	+	-	-	-	-	+	-	Alive at 35y	
4	F	1.5 m	/	+	+	+	+	-	+	-	-	-	-	+	-	Alive at 28y	
5	M	2 m	/	+	+	+	-	-	+	-	-	-	-	+	-	Dead at 4y after fever	
6	M	1 m	/	+	+	+	+	-	+	-	+	-	-	+	+	Alive at 6y	
7	M	3 m	/	+	+	+	+	-	+	-	+	-	-	+	-	Dead at 8y after fever	
8	M	1 m	/	+	+	+	+	-	+	-	-	-	+	+	+	Alive at 1.7y	
9	M	2y	/	+	+	+	+	-	+	-	-	-	-	+	-	Alive at 5y	
10	F	5 m	/	+	+	+	+	+	+	-	-	-	-	+	-	Alive at 7y	
11	M	1.4y	-	+	+	+	-	-	-	-	-	-	-	+	/	Alive at 4y	[6]
12	F	0 d	+	+	+	+	-	-	-	-	+	-	-	+	/	Dead at 2 m with MODS	[7]
13	F	5.5y	/	+	+	+	+	-	+	-	-	-	-	+	+	Alive at 5.5y	[8]
14	F	3 m	-	+	+	+	-	-	-	-	-	-	-	+	+	Alive at 5 m	[9]
15	F	/	+	+	+	/	/	+	+	/	+	/	/	+	+	Alive at 1.3y	[10]
16	F	4y	+	+	/	+	+	+	+	-	-	-	+	+	-	Alive at 12y	[5]
17	M	1y	-	+	/	+	+	-	-	-	-	-	+	+	+	Alive at 6y	
18	M	1y	+	+	+	+	+	+	+	-	-	-	+	+	+	Alive at 8y	
19	M	3.5 m	-	+	+	+	+	+	+	-	-	-	+	+	/	Dead at 1.8y after fever	
20	F	23y	+	+	+	+	+	+	+	-	-	-	+	+	+	Alive at 23y	
21	F	3y	-	/	+	-	+	-	-	-	-	-	+	/	/	Alive at 6y	
22	M	5y	-	/	+	+	+	-	+	-	-	-	+	/	/	Alive at 8y	
23	M	11 m	+	+	+	+	+	-	+	-	-	-	+	+	+	Alive at 12y	
24	M	1.2 m	+	+	+	+	+	+	+	-	-	-	-	+	+	Liver transplanted at 4 m	
25	F	1y	+	+	+	+	+	+	+	-	-	-	+	+	+	Alive at 3y	
26	F	1.2 m	+	+	+	+	+	-	-	-	-	-	+	+	-	Alive at 1y	
27	M	5 m	+	+	+	+	+	-	-	-	-	-	+	+	-	Alive at 1y	
28	M	0 d	+	+	/	+	-	-	-	-	-	-	+	+	-	Dead at 2 m with MODS	[11]
29	M	0 d	+	+	/	+	+	-	+	-	+	-	-	+	/	Dead at 3 m with MODS	[3]
30	M	4 m	-	+	+	+	+	-	-	-	-	-	-	+	/	Alive at 3y	[12]
31	M	2 m	-	+	+	+	-	-	-	-	-	+	+	+	-	Alive at 6 m	[13]
32	M	0.2 m	/	/	/	/	/	/	/	/	/	/	/	/	/	Dead	[14]
33	/	1 m	/	/	+	/	/	+	+	/	/	/	/	/	/	Dead at 5 m	[15]
34	M	0 d	+	+	+	+	+	-	-	+	-	-	+	+	-	Dead at 8.4 m after fever	Our study
35	M	1 m	-	-	-	+	-	-	-	-	-	-	-	+	+	Dead at 2 m with diarrhea	
36	F	0 d	+	+	+	+	-	-	-	-	-	-	-	+	(-)	Dead at 6 m after fever	

*Abbreviations*: *G* Gender, *M* Male, *m* month, *F* Female, *y* year, *d* day, *Ref* Referenced publication, *IUGR* Intrauterine growth retardation, *FTT* Failure to thrive, *NS* Nervous system, *+* present, - absent, / data not available or data not reported, *MODS* Multiple organ dysfunction syndrome

The main clinical signs presented by the patients included failure to thrive (30/31), anaemia (32/33), and hypoalbuminemia (32/32). Furthermore, 34 out of 36 patients had liver involvement, including 3 out of 34 with coagulation abnormalities, 17 out of 34 with acute liver failure, 7 out of 34 with recurrent liver failure, 9 out of 12 with jaundice, and 32 out of 34 with elevated enzymes. Additionally, 28 out of 35 patients exhibited nervous system involvement, including 25 out of 30 with mental retardation, 9 out of 18 with encephalopathy, and 22 out of 29 with seizures. Fifteen out of 18 patients experienced hepatomegaly and/or splenomegaly, while 7 out of 34 patients had renal involvement, with 1 patient having renal calculus, 5 having kidney failure, and 1 having renal tubular injury. Muscle involvement was also present in 15 out of 31 patients, with 13 out of 27 having hypotonia and 2 out of 4 having skeletal muscle abnormalities. Hypoglycemia was reported in 12 out of 27 patients, and hyperammonemia was reported in 6 out of 28 patients. Finally, a chubby face was observed in 2 out of 4 patients. These findings are summarized in Table 2 and Table S1.

There were 11 cases that died between the ages of 2 months and 8 years. One patient survived at 3 years during last follow-up after undergone liver transplantation for acute liver failure at the age of 4 months. Seven cases died due to multiple organ failure induced by liver failure before reaching the age of 1 year. Three cases died due to infection-related or associated encephalopathy at the ages of 20 months, 4 years, and 8 years respectively. Among the 24 children whom survived with their own native liver at the last follow-up, 18 had a follow-up age larger than 3 years, but another 6 children had no follow-up information at the age of 3 years. In addition, it is interesting to note that cases older than 2 years exhibited repeated elevations of transaminase, but no cases of liver failure were observed.

### Genetic features of ILFS1 patients

Whole exon sequencing indicated biallelic variation in the *LARS1* gene was observed in the three cases identified in our medical center, i.e., Case1: c.1284G > A (p.Pro428Pro) / c.3379 C > T (p.Arg1127Ter); Case 2: c.497T > C (p.Leu166Pro) / c.2806T > C (p.Cys936Arg); Case 3: c.1321 C > T (p.Arg441Ter) / c.149 C > G (p.Ala50Gly)]. Six novel variants were identified in the three new patients, including two nonsense variants, three missense variants and one synonymous variant. The pathogenicity of the newly discovered missense *LARS1* variants were assessed using in silico tools (Tables 3 and 4). Of the reported cases, 32 had complete genetic data and 34 variants, including frameshift variants, nonsense variants, splice site variants, and missense variants, were identified. The genetic mutations of c.1118 A > G (p.Tyr373Cys), c.245 A > G (p.Lys82Arg), c.1292T > A (p.Val431Asp) and c.3313 C > T (p.Arg1105Ter) were identified in thirteen, nine, eight and four alleles, respectively. The mutation of c.3420del (p.Ile1141PhefsTer12) was found in three alleles, while the mutations of c.1283 C > T (p.Pro428Leu), c.1838_1843del (p.Gly613_Leu615delinsVal), c.2445G > T (p.Met815Ile), and c.587G > C (p.Gly196Ala) each occurred two times (Table 4). However, no significant correlations were observed between the type of variants and the presence of liver failure or the severity of diseases among reviewed patients (Supplementary Material 1, Supplementary Table S1).

**Table 3:** *LARS1* variants, hepatic manifestations, and liver biopsy findings of reported ILFS1 patients

Basis Information		LARS1 variant		Liver				liver biopsy			Ref
ID	nation	NM_020117.11		abnormal trans-aminase	jaundice	liver failure	hepatosplenomegaly	steatosis	fibrosis	cirrhosis	
1	Irish Traveller	c.245 A > G; *p*.Lys82Arg	c.1118 A > G; *p*.Tyr373Cys	+	/	++	/	+	+	+	[1]
2	Irish Traveller	c.245 A > G; *p*.Lys82Arg	c.1118 A > G; *p*.Tyr373Cys	+	/	++	/	/	/	/	
3	Irish Traveller	c.245 A > G; *p*.Lys82Arg	c.1118 A > G; *p*.Tyr373Cys	+	/	-	+	/	/	/	
4	Irish Traveller	c.245 A > G; *p*.Lys82Arg	c.1118 A > G; *p*.Tyr373Cys	+	/	-	+	/	/	/	
5	Irish Traveller	c.245 A > G; *p*.Lys82Arg	c.1118 A > G; *p*.Tyr373Cys	+	/	+	/	+	+	+	
6	Irish Traveller	c.245 A > G; *p*.Lys82Arg	c.1118 A > G; *p*.Tyr373Cys	+	/	-	/	/	/	/	
7	Irish Traveller	c.245 A > G; *p*.Lys82Arg	c.1118 A > G; *p*.Tyr373Cys	+	/	+	/	/	/	/	
8	Irish Traveller	c.245 A > G; *p*.Lys82Arg	c.1118 A > G; *p*.Tyr373Cys	+	/	++	/	/	/	/	
9	Irish Traveller	c.245 A > G; *p*.Lys82Arg	c.1118 A > G; *p*.Tyr373Cys	+	/	-	/	/	/	/	
10	Ashkenazi Jewish	c.1511 C > T; *p*.Ala504Val	c.1842 C > G; *p*.Asn614Lys	+	/	+	/	+	-	-	
11	China	c.2133_2135del; p.Leu712del	c.1183G > A; p.Asp395Asn	+	-	+ / -	+	+	+	+	[2]
12	Caucasian	c.725 C > T; p.Pro242Leu	c.1292T > A; p.Val431Asp	+	+	+	-	/	/	/	[3]
13	/	c.1283 C > T; p.Pro428Leu	c.3420del; p.Ile1141PhefsTer12	+	/	-	+	+	+	+	[4]
14	China	c.2422delA; p.Thr808fs	c.478 A > G; p.Ile160Val	+	+	+ / -	+	/	/	/	[16]
15	/	N/A	N/A	/	/	++	+	/	/	/	[5]
16	Germany	c.1292T > A; p.Val431Asp	c.641 A > G; p.Tyr214Cys	+	/	++	/	+	-	-	[6]
17	Germany	c.1292T > A; p.Val431Asp	c.2445G > T; p.Met815Ile	+	/	+	/	-	+	+	
18	Germany	c.1292T > A; p.Val431Asp	c.1284 + 1delG	+	/	+	/	-	+	+	
19	Netherlands	c.3420del; p.Ile1141PhefsTer12	c.587G > C; p.Gly196Ala	+	/	+	/	-	-	-	
20	Netherlands	c.3420del; p.Ile1141PhefsTer12	c.587G > C; p.Gly196Ala	+	/	+/-	/	/	/	/	
21	Syrian Arab Republic	c.3313 C > T; p.Arg1105Ter	c.3313 C > T; p.Arg1105Ter	-	-	-	-	/	/	/	
22	Syrian Arab Republic	c.3313 C > T; p.Arg1105Ter	c.3313 C > T; p.Arg1105Ter	-	-	-	-	/	/	/	
23	France	c.1292T > A; p.Val431Asp	c.644 A > C; p.Asp215Ala	+	/	++	/	-	+	-	
24	USA	c.2003T > A; p.Val668Asp	c.2445G > T; p.Met815Ile	+	/	+	/	-	+	-	
25	Germany	c.1292T > A; p.Val431Asp	c.1838_1843del; p.Gly613_Leu615delinsVal	+	/	+	/	/	/	/	
26	Germany	c.1292T > A; p.Val431Asp	c.1838_1843del; p.Gly613_Leu615delinsVal	+	/	-	+	/	/	/	
27	Ireland	c.1118 A > G; p.Tyr373Cys	c.1118 A > G; p.Tyr373Cys	+	/	+	/	/	/	/	
28	Japan	c.1351 A > T; p.Leu451Phe	c.213 + 1G > A	+	+	+	+	-	+	-	[7]
29	Italy	c.1283 C > T; p.Pro428Leu	c.743G > T; p.Cys248Phe	+	+	+	/	-	+	-	[8]
30	China	c.1367 A > G; p.Asp456Glu	c.2212 + 1 A > G	+	+	+	+	/	/	/	[9]
31	France	c.463 A > G; p.Lys155Glu	c.1818dup; p.Ala607Cysfs*8	+	+	+	+	/	/	/	[10]
32	/	c.1292T > A; p.Val431Asp	c.312G > T; p.Leu104Phe	/	/	+	/	/	/	/	[11]
33	/	c.1159G > A; p.Gly387Ser	c.1159G > A; p.Gly387Ser	+	/	/	+	/	/	/	[12]
34	China	c.1284G > A; p.Pro428Pro	c.3379 C > T; p.R1127Ter	+	+	++	+	/	/	/	Our study
35	China	c.1321 C > T; p.Arg441Ter	c.149 C > G; p.Ala50Gly	+	+	+	+	+	-	-	
36	China	c.497T > C; p.Leu116Pro	c.2806T > C; p.Cys936Arg	+	+	+	+	/	/	/	

*Abbreviations*: *Ref* Referenced paper, + positive, - negative, / data not available or data not reported, *+/- in liver failure column* with coagulation abnormality, *+ in liver failure column* with liver failure, *++ in liver failure column* with recurrent liver failure

**Table 4:** Allele frequencies and *in silico* prediction of 4 novel *LARS1* missense/ synonymous variants

cDNA change (NM_020117.11)	Protein change (NP_000383.2)	gnomAD	gnomAD_EAS	MuT	SIFT	PROVEAN	M-CAP	FATHMM	REVEL
c.1284G > A	p.Pro428Pro	0.000028 (0/8/282,094)	0 (0/0/19,944)	D	/	/	/	/	/
c.149 C > G	p.Ala50Gly	-	-	D	D	D	D	D	D
c.497T > C	p.Leu116Pro	-	-	D	D	D	D	N	N
c.2806T > C	p.Cys936Arg	-	-	D	N	N	D	N	N

*Abbreviations:* *gnomAD and gnomAD_EAS* allele frequencies of corresponding variants in all populations and in East Asian populations in gnomAD (http://gnomad-old.broadinstitute.org/) respectively, *D* disease-causing, / not applicable, - not reported, *N* not effect, *MuT* MutationTaster (http://www.mutationtaster.org), *SIFT* Sorting Intolerant From Tolerant (http://provean.jcvi.org/index.php), *PROVEAN* Protein Variation Effect Analyzer (http://provean.jcvi.org/index.php), *M-CAP* Mendelian Clinically Applicable Pathogenicity (http://bejerano.stanford.edu/MCAP/), *FATHM* Functional Analysis Through Hidden Markov Models (http://fathmm.biocompute.org.uk/), *REVEL* Rare Exome Variant Ensemble Learner (https://sites.google.com/site/revelgenomics/

### Pathological findings

**Fig. 2:**
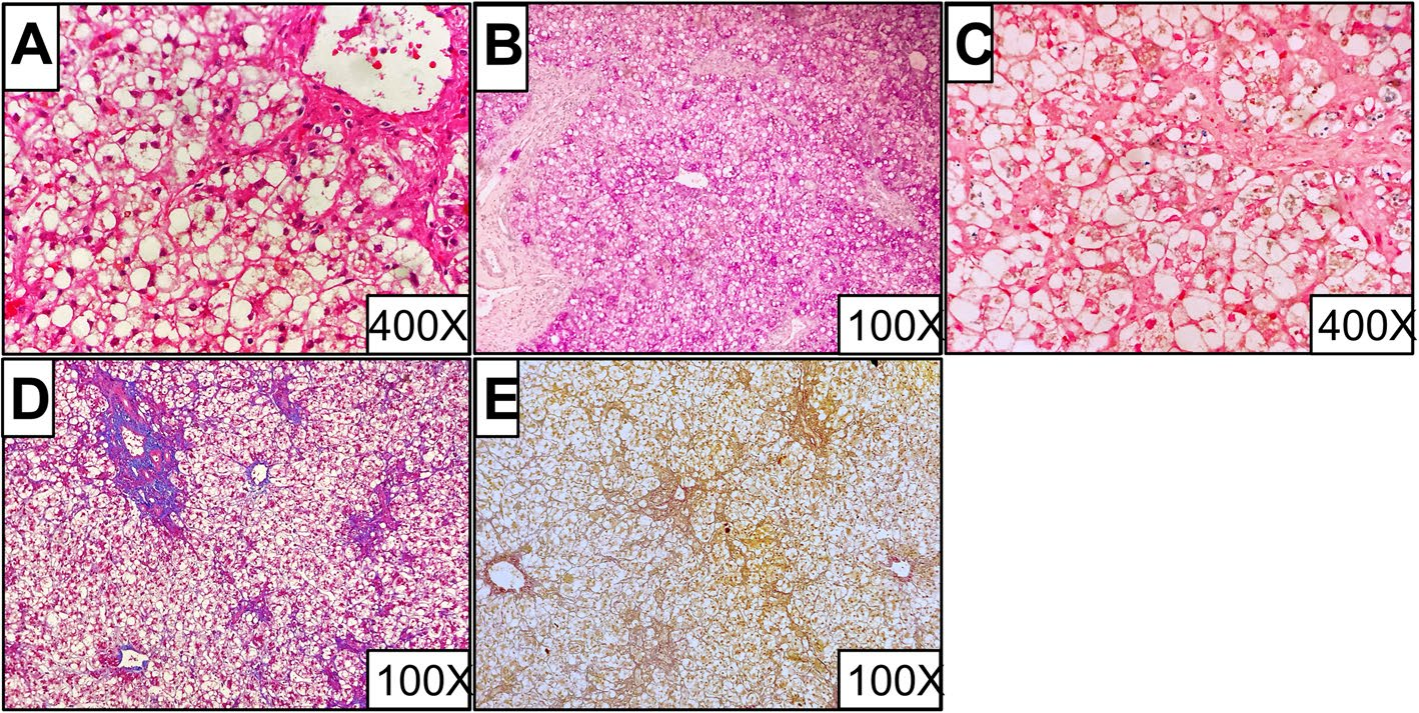
Histopathologic features, liver biopsy specimen, Patient 3. Ballooning and steatosis characterize hepatocytes, with focal rosetting. Flecks of bile pigment are seen in hepatocyte cytoplasm, as are occasional canalicular bile plugs. Stainable iron is present in Kupffer cells. Portal-tract fibrosis is mild to moderate, with perisinusoidal extension, spurring, and suggestions of early portal – portal bridging. **A** – **E**, respectively hematoxylin and eosin; periodic acid – Schiff technique; Perls’ stain for iron; Masson’s trichrome stain; and reticulin stain. For original magnifications, see individual images

Out of the three new cases, liver biopsy specimens were obtained from case 3 by needle biopsy. The biopsy of this case showed hepatocyte diffuse steatosis, cholestasis, fibrosis, and regional iron deposition, as displayed in Fig. 2. No liver biopsies were performed for case 1 and case 2 due to liver failure that occurred during hospitalization. Out of the 33 reported cases, 14 showed pathological findings and the common manifestations included steatosis (7/14) with mixed bulla and vesicles in some of these cases, liver fibrosis (10/14) and cirrhosis (6/14) (See Table 3).

**Fig. 3:**
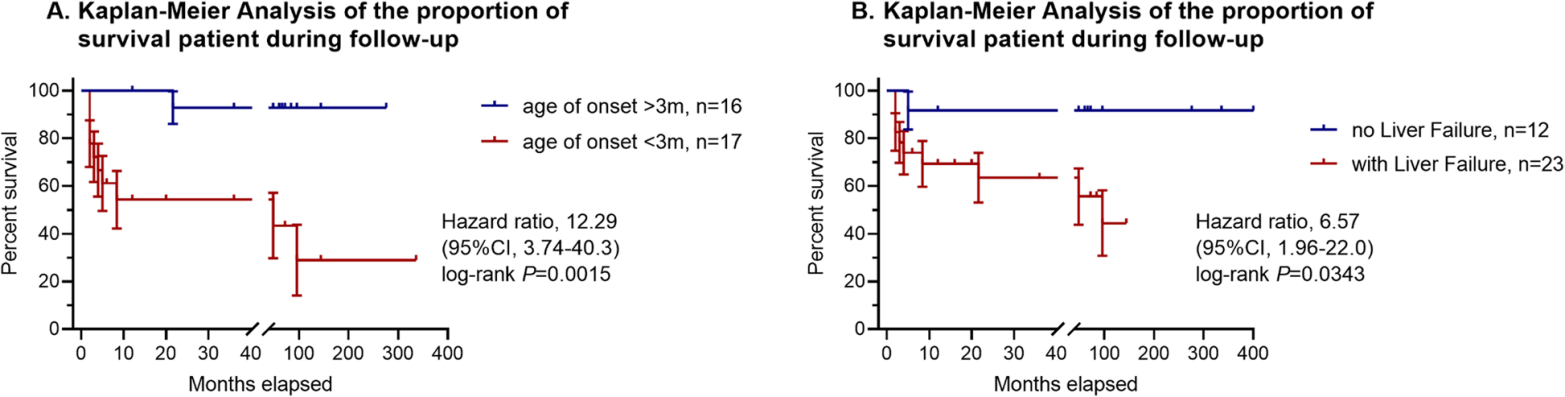
Kaplan-Meier plotter analyse: **A** the early age of onset (less than 3 months) and **B **the occurrence of liver failure was associated with poor prognosis (died or underwent liver transplantation)

Kaplan-Meier plotter analysis indicated that early age of onset and occurrence of liver failure were the main factors associated with the prognosis of patients. We used the Kaplan-Meier method to perform survival analysis and assessed the association between patients’ demographic data, such as the child’s gender and age of onset (before or after 3 months), and the main clinical manifestations. The primary clinical manifestations that were considered included liver failure, premature delivery, failure to thrive, anemia, hypoalbuminemia, nervous system involvement, hepatomegaly and/or splenomegaly, renal involvement, muscle involvement, hypoglycemia, and hyperammonemia. We also analyzed patients’ follow-up information, such as age at death or liver transplantation, censoring during follow-up, or their last clinical contact as recorded. As a result, no significant correlation between the prognosis of patients and the clinical curse of patients was found except for the age of onset (*P* = 0.0015, Hazard ratio = 12.29, 95% CI = 3.74–40.3) and the occurrence of liver failure (*P* = 0.0343, Hazard ratio = 6.57, 95% CI = 1.96-22.0) (Fig. 3, other data were not shown).

## Discussion

ILFS1 is a life-threatening rare disease. Since the initial description of 10 patients in 2012 [1], only 23 new ILFS1 cases have been published. This report describes 3 new instances of ILFS1 in non-Irish children, all of whom died before 1y of age. Review of the 36 patients reported to date found onset of ILFS1 to range from first postnatal day to 23y (median 2mo) and was at age < 3mo in 17/36 patients.

Principal features of ILFS1 were IUGR (31/32, not addressed in 4 case descriptions), premature birth (14/23, not addressed in 13), failure to thrive (30/31, not addressed in 5), hepatomegaly and/or splenomegaly (15/18, not addressed in 18), kidney involvement (7/34, not addressed in 2), neurodevelopmental delay (25/30, not addressed in 6), seizures (22/29, not addressed in 7), and muscular hypotonia (13/27, not addressed in 9). In addition, microcytic anemia (32/33, not addressed in 3), hypoalbuminemia (32/32, not addressed in 4), acute liver failure (24/34, not addressed in 2), and hypoglycaemia (12/27, not addressed in 9) were encountered. Of possible note in our patients were a chubby face and loose skin in patient 1, reminiscent of an earlier description [16], and persistent diarrhea in patient 3.

Liver disease is a principal site of involvement in ILFS1. Biomarker values and clinical evidence of hepatobiliary disease varied among 34 individuals (the other 2 cases have no liver involvement) in whose cases data were available, with only elevated serum transaminase activities and full-blown liver failure in others. Liver failure is the most dramatic sign of *LARS1* disease; it can be life-threatening. Indeed, in 7 cases death in multiple organ failure complicating liver failure ensued before age 1y, and another patient survived after underwent liver transplantation aged 4mo for acute liver failure. Recurrent liver failure has been observed, particularly in association with fever [5]. Risk of liver failure appears to fall in older patients, however: No instance was reported in patients aged > 2y, with only elevations in serum transaminase activities observed thereafter, abnormalities that resolved entirely by age 16y [5]. While liver-biopsy features in 14 patients were non-specific, steatosis (7/14), fibrosis (10/14), and cirrhosis (6/14) were frequent.

The nervous system is also prominently involved. Principal manifestations included neurodevelopmental retardation (25/30, not addressed in 9), epilepsy (22/29, not addressed in 7), and infection-related encephalopathy (9/18, not addressed in 18). Death occurred during *LARS1* related encephalopathy, associated with intercurrent febrile infection, at ages 20mo, 4y, and 8y. Whilst liver failure is more common before age < 1y, nervous-system involvement is more frequent in older children (perhaps because nervous-system involvement is then more readily recognizable). One child who underwent liver transplantation for acute liver failure aged 4mo on follow-up despite normal liver function exhibited delayed mental development and experienced encephalopathy [5]. Further observations are required to determine if liver transplantation is appropriate in *LARS1* disease, given multisystem impairment, especially with nervous-system involvement. Despite delays in early neurodevelopment, however, follow-up assessment in an Irish traveller cohort found positive neurodevelopment in most patients (all patients attend mainstream education except one which attends a special need school) during follow up [5].

Complete genetic data were obtained for 35 patients and 6 novel variants were identified in the three new patients. We have found that the mutations of c.1118 A > G (p.Tyr373Cys), c.245 A > G (p.Lys82Arg), c.1292T > A (p.Val431Asp), c.3313 C > T (p.Arg1105Ter), and c.3420del (p.Ile1141PhefsTer12) occurred multiple times (Table 3), suggesting that these are frequent variants in the population. No significant correlations between patient genotype and prognosis were found in the study cohort (Supplementary Material 1 and Supplementary Table S1). This could be due to the limited number of cases. Furthermore, it is reasonable to assume that there are other factors that impact the disease phenotype.

The fact that one-third (12/36) of the patients died or underwent liver transplantation between the ages of 2 months and 8 years indicates a poor prognosis of the disease. In the current study, we used Kaplan-Meier plotter analysis to assess the main factors that may be associated with the patients’ prognosis. We have found that the early age of onset (less than 3 months) and the occurrence of liver failure are the main factors associated with the poor prognosis. Both P values were less than 0.05. However, we did not find other significant correlations between the prognosis of patients and the clinical course, such as nervous system involvement or kidney involvement. This may be due to the limited cases, which hampers the statistical significance. For example, there are only a few patients without nervous system involvement or kidney involvement. However, we believe that the current data could still be significant for caregivers who are pursuing better outcome for the patients. It is important to provide intensive care to the patients with an early onset of liver failure in order to restore normal liver function, which may occur either through treatment or as the children grow up.

The pathogenic mechanism behind *LARS1* gene defects remains not fully understood. There are limited therapeutic options available for patients with ILFS1. However, among the 12 children with a poor prognosis, 5 died after experiencing fever. It appeared that fever may be a common triggering factor for acute liver failure and encephalopathy in ILFS1 patients, which can lead to death. Lenz [5] has found that the aminoacylation activity of cells in all patients was significantly decreased when the temperature was increased in vitro. This might explain the susceptibility of *LARS1* gene defect to induce acute liver failure and encephalopathy in the case of fever. Early antipyretic therapy and infection prevention are essential for improving clinical outcomes in patients with ILFS1. Regular vaccinations may also reduce the incidence of infection. If an infection occurs, it is recommended that the patient be admitted to the hospital, provided with close observation, and given nutritional support. This is because the patient may experience lethal acute liver failure and encephalopathy during the course of the disease. Additional patients with longer follow-up are required to investigate whether taking active precautions against fever could improve the prognosis of these patients.

## Conclusions

The ILFS1, which is caused by *LARS1* variants, is a rare and life-threating disease. In the current study, we have summarized the clinical manifestations, genetic features, and pathological findings of reported cases. Our findings indicated that an early onset of age and the occurrence of liver failure are associated with a poor prognosis.

### Supplementary Information

Supplementary Material 1.

Supplementary Material 2.

## Data Availability

All data generated or analysed during this study are included in this published article and its supplementary information files.

## Abbreviations

ALB: Albumin
ALT: Alanine aminotransferase
AST: Aspartate aminotransferase
CI: Confidence interval
DB: Direct bilirubin
GGT: Gamma-glutamyl transferase
HB: Hemoglobin
ILFS1: Infant liver failure syndrome type 1
INR: International normalized ratio
IQR: Interquartile range
IUGR: Intrauterine growth retardation
LARS1: Leucyl-tRNA synthase 1
MODS: Multiple organ dysfunction syndrome
NRM: Normal reference range
TB: Total bilirubin
TBA: Total bile acid
tRNA: Aminoacylation of transfer RNA
UDCA: Ursodeoxycholic acid
