# Supplementary material for: Early onset and liver failure indicating poor prognosis of infant liver failure syndrome type 1
Source: Orphanet J Rare Dis. 2024 Jun 6;19:225. doi: 10.1186/s13023-024-03229-3 (PMC11155007; doi:10.1186/s13023-024-03229-3)
Supplement: Supplementary file 1 — Supplementary Material 1. [file 13023_2024_3229_MOESM1_ESM.docx]

# Supplementary Table S1. Correlation of genotype and phenotype in patients with LARS1 variants

| Phenotype groups | With null variants | | Biallelic non-null variants | Total | Analysis |
| --- | --- | --- | --- | --- | --- |
|  | Biallelic null variants | Single null variant |  |  |  |
| with liver failure group | 1 | 4 | 6 | 11 | P=0.459 |
| no liver failure group | 0 | 7 | 16 | 23 |  |
| Severe | 0 | 4 | 5 | 9 | P=1.000 |
| Mild | 2 | 4 | 13 | 19 |  |
