# Supplementary figures and images for: Early onset and liver failure indicating poor prognosis of infant liver failure syndrome type 1
Source: Orphanet J Rare Dis. 2024 Jun 6;19:225. doi: 10.1186/s13023-024-03229-3 (PMC11155007; doi:10.1186/s13023-024-03229-3)

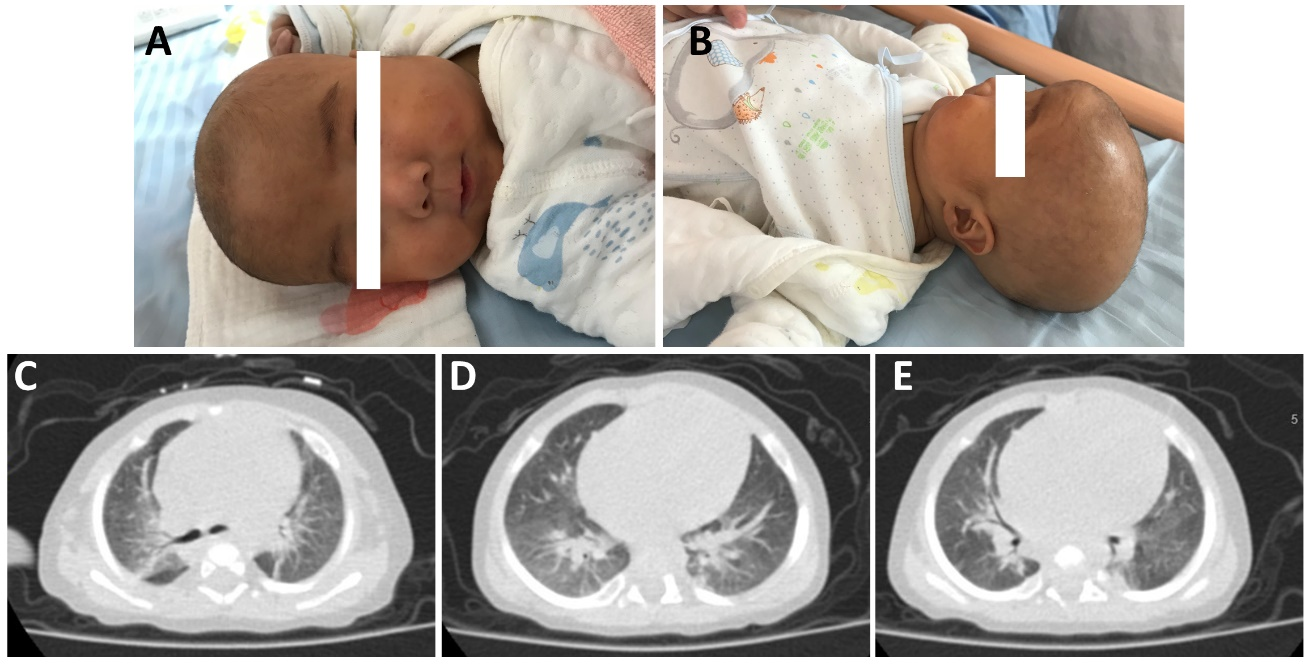

Supplement: Supplementary file 2 — Supplementary Material 2. [file 13023_2024_3229_MOESM2_ESM.tiff]
